# Evoked EEG Responses to TMS Targeting Regions Outside the Primary Motor Cortex and Their Test–Retest Reliability

**DOI:** 10.1007/s10548-023-01018-y

**Published:** 2023-11-23

**Authors:** Yufei Song, Pedro C. Gordon, Johanna Metsomaa, Maryam Rostami, Paolo Belardinelli, Ulf Ziemann

**Affiliations:** 1 Department of Neurology and Stroke, University of Tübingen, Hoppe-Seyler-Straße 3, 72076 Tübingen, Germany; 2 Hertie Institute for Clinical Brain Research, University of Tübingen, Tübingen, Germany; 3 Department of Neuroscience and Biomedical Engineering, Aalto University School of Science, Espoo, Finland; 4 Faculty of Electrical and Computer Engineering, University of Tehran, Tehran, Iran; 5 Center for Mind/Brain Sciences, CIMeC, University of Trento, Trento, Italy

**Keywords:** Transcranial magnetic stimulation, Electroencephalography, TMS-EEG, Optimized sham procedure, Evoked potentials, Test–retest reliability

## Abstract

Transcranial magnetic stimulation (TMS)–evoked electroencephalography (EEG) potentials (TEPs) provide unique insights into cortical excitability and connectivity. However, confounding EEG signals from auditory and somatosensory co-stimulation complicate TEP interpretation. Our optimized sham procedure established with TMS of primary motor cortex (Gordon in JAMA 245:118708, 2021) differentiates direct cortical EEG responses to TMS from those caused by peripheral sensory inputs. Using this approach, this study aimed to investigate TEPs and their test–retest reliability when targeting regions outside the primary motor cortex, specifically the left angular gyrus, supplementary motor area, and medial prefrontal cortex. We conducted three identical TMS–EEG sessions one week apart involving 24 healthy participants. In each session, we targeted the three areas separately using a figure-of-eight TMS coil for active TMS, while a second coil away from the head produced auditory input for sham TMS. Masking noise and electric scalp stimulation were applied in both conditions to achieve matched EEG responses to peripheral sensory inputs. High test–retest reliability was observed in both conditions. However, reliability declined for the ‘cleaned’ TEPs, resulting from the subtraction of evoked EEG response to the sham TMS from those to the active, particularly for latencies > 100 ms following the TMS pulse. Significant EEG differences were found between active and sham TMS at latencies < 90 ms for all targeted areas, exhibiting distinct spatiotemporal characteristics specific to each target. In conclusion, our optimized sham procedure effectively reveals EEG responses to direct cortical activation by TMS in brain areas outside primary motor cortex. Moreover, we demonstrate the impact of peripheral sensory inputs on test–retest reliability of TMS-EEG responses.

**Supplementary Information:**

The online version contains supplementary material available at 10.1007/s10548-023-01018-y.

## Introduction

Transcranial magnetic stimulation (TMS) is a non-invasive technique that can activate neuronal circuits in the cortex via an induced electric field (Barker et al. 1985). Simultaneous electroencephalography (EEG) can be combined with TMS to probe cortical responsivity to stimuli with millisecond-level temporal resolution (Ilmoniemi et al. 1997). TMS-evoked potentials (TEPs) are one of the resulting outcomes, and they are time-locked deflections that demonstrate high consistency and responsiveness to variations in the stimulation parameters, e.g., TMS intensity and cortical locations (Bortoletto et al. 2015; Tremblay et al. 2019). TEPs are hypothesized to reflect the effects of TMS on the local neural circuits and networks, which suggests their application as a measure of cortical excitability and connectivity. However, a recent discussion has arisen about the extent to which TEPs truly reflect the direct cortical activation induced by TMS (Belardinelli et al. 2019; Conde et al. 2019; Siebner et al. 2019). This is because the spatiotemporal patterns of TEPs resemble those of peripheral evoked potentials (PEPs), which are evoked by, e.g., electric stimulation (ES) applied to the scalp, or TMS applied to the shoulder (Conde et al. 2019; Herring et al. 2015). Findings from studies using PEP controlling approaches have reinforced the TEPs' capability to reflect direct cortical activation (Biabani et al. 2019; Gordon et al. 2021, 2023; Rocchi et al. 2021). However, supporting evidence for TMS of cortical regions outside the primary cortex (M1) is still limited.

Proper control of PEPs is essential for revealing direct cortical response to TMS and avoiding potential misinterpretation of TEPs. Nevertheless, achieving this can be challenging. PEPs are the cortical responses to multisensory inputs that are not directly related to the transcranial effects of TMS but still exhibit a time-locked relationship with it (Farzan and Bortoletto 2022; Hernandez-Pavon et al. 2023). The two main contributing EEG sources to PEPs are the auditory-evoked potentials (AEPs) and the somatosensory-evoked potentials (SEPs). The former is caused by the ’click’ sound of the TMS pulse, which reaches the participant’s inner ears through air and bone conduction. The latter is generated by multiple sources, including coil vibration and cranial muscle twitches. The activation of cranial sensory and motor axons, nerve bundles, and peripheral sensory receptors is believed to play a role (Siebner et al. 2022). To date, the approaches available to control these confounding factors have remained limited. For instance, masking noise (Massimini et al. 2005; Russo et al. 2022) has been commonly used during TMS–EEG data recording, aiming to minimize the AEPs by suppressing the auditory perception. However, complete effectiveness cannot be guaranteed when high TMS intensities are required as the louder ’click’ sound from the TMS pulse may overwhelm the masking noise (Conde et al. 2019). Moreover, even when an optimal masking procedure is implemented (masking noise through earphones plus over-ear defender), TMS–EEG measurements are still at risk of presenting PEPs due to the effects of somatosensory inputs (Conde et al. 2019; Ross et al. 2022). Proposed approaches to deal with the confounding of SEPs include using foam padding to reduce coil vibration, limiting cortical targets to regions close to the midline where cranial muscle artifact is minimal, and online fine-tuning stimulation parameters to minimize artifacts (Casarotto et al. 2022). These strategies certainly offer some benefits, but each has its caveats, and none is reliable enough to address the contamination by PEPs sufficiently.

An alternative approach is to apply a sham condition that recreates the same multisensory inputs expected from TMS without transcranially activating the cortex. In this instance, the contribution of sensory inputs to the TMS–EEG responses can be determined by comparing the results between active and sham conditions. Sham TMS conditions often include a second TMS coil placed away from the scalp to produce a ’click’ sound and scalp ES to simulate TMS-associated somatosensory inputs (Conde et al. 2019; Fernandez et al. 2021; Raffin et al. 2020; Rocchi et al. 2021). In principle, PEPs generated in the sham TMS condition can be subtracted from, or statistically compared to, the EEG responses elicited by the active TMS condition, thus revealing the EEG responses to direct cortical activation by TMS. However, since the somatosensory inputs related to TMS are qualitatively different from cutaneous ES, one drawback of this design is the potential mismatch of PEPs elicited by sham and active TMS conditions. To mitigate this issue, our group introduced an optimized sham stimulation procedure: By delivering high-intensity ES during both active and sham TMS pulses, we aimed to saturate the EEG response to sensory inputs. Consequently, the additional sensory input from TMS in the active condition is negligible, resulting in matched PEPs from the sham and the active TMS conditions. We successfully tested this approach using TMS targeting the left M1. After removing PEPs from the evoked EEG responses in the active TMS condition, we identified typical deflections lateralized to the TMS site within the first hundred milliseconds after the TMS pulse (Gordon et al. 2021).

Here, we sought to utilize this approach further to investigate the TMS evoked EEG responses of regions outside M1. To this end, three cortical areas, the left angular gyrus (AG), supplementary motor area (SMA) and medial prefrontal cortex (mPFC) were targeted. These areas are known to be involved in various cognitive and motor functions and have gained growing interest in TMS studies as possible targets for therapeutic interventions (Gordon et al. 2022; Lefaucheur et al. 2020; Seghier 2023). In the present study, participants underwent three identical sessions at least one week apart. Within each session, the three cortical areas were targeted separately, and an optimized sham procedure adapted from our previous study was applied (Gordon et al. 2021). As outcomes, we investigated the test-retest reliability of TMS–EEG responses between repeated sessions. Then, the EEG responses elicited by the active TMS were compared with those from the sham TMS condition for all TMS targets. We hypothesized that, using this approach, differences in EEG responses between the active and sham TMS conditions could be identified, which would reflect the target area specific characteristics of direct cortical activation caused by TMS.

## Methods

### Participant and General Procedure

Twenty-four healthy participants were recruited (mean age ± SD, 25.7 ± 4.8 years; 14 females) for the study. Inclusion criteria were no history of neurological or psychiatric disorders and no intake of medication acting on the central nervous system. The experimental procedures were approved by the ethics committee of the medical faculty of Tübingen University (protocol number 638/2020BO1). All participants provided written informed consent at enrollment in accordance with the declaration of Helsinki.

Prior to the main measurements, magnetic resonance imaging (MRI) with a T1-weighted sequence was obtained for each participant. The individualized MRI was used for TMS neuronavigation, EEG electrode location pin-pointing, and EEG source reconstruction. The main experiment consisted of three sessions (at least one week apart). During each session, participants’ resting motor threshold (RMT) was determined, and then they underwent three TMS–EEG blocks. In each block, either mPFC, AG, or SMA was targeted, and the order of the blocks was randomized for each participant and session. Both sham and active TMS were conducted for every block (see details in section “Optimized sham TMS procedure”). Participants were instructed to sit on a chair in a relaxed and still position with their eyes open throughout the measurements. To ensure head stability and comfort, a vacuum pillow was fitted around the neck. At the beginning of each TMS–EEG block during the first session, participants were asked to rate their perception of auditory and somatosensory inputs from the active and sham conditions (Gordon et al. 2021). This was done using a visual analog scale (VAS) ranging from 0 to 10, with 0 representing no perception and 10 representing maximal perception. The VAS included items assessing the intensity of auditory sensation, the intensity of scalp sensation, the area size of scalp sensation, and the intensity of pain or discomfort.

### TMS–EEG Setup

Two identical TMS stimulators (Magstim® 200^2^, monophasic mode, UK) equipped with figure-of-eight coils (external diameter = 90 mm) were used in the study (one for active TMS condition, one for sham condition). A navigation system (TMS Navigator, Localite GmbH, Germany) was used for planning and monitoring coil positioning throughout the measurement. We aligned the individual MRI with the MNI coordinate system, and the cortical targets were defined with the following MNI coordinates: mPFC (− 4, 52, 36); left AG (− 42, − 69, 31); SMA (− 2, − 7, 55). The active TMS coil was positioned perpendicularly to the underlying gyrus, with the induced current running approximately from lateral to medial for targeting mPFC and SMA, and from posterior-medial to anterior-lateral for TMS at AG. We set the intensity for each target in active TMS conditions to 120% RMT. The RMT was measured in a standard manner (Groppa et al. 2012): bipolar surface Electromyographic (EMG) electrodes were attached to the abductor pollicis brevis (APB), and first dorsal interosseus (FDI) muscles of the right-hand, EMG signals were sampled at 5 kHz (device filter DC-1250 Hz). The hotspot for M1 was determined as the cortical representation of the hand eliciting the largest and consistent motor-evoked potential (MEP) with slightly suprathreshold TMS pulses. The RMT was defined as the lowest intensity that produced MEP over 50 µV in at least 5 out of 10 pulses.

We recorded EEG signals with a TMS-compatible system (NeurOne, Bittium, Kuopio, Finland). Ag/AgCl sintered ring electrodes were placed according to the International 10-5 system in an elastic cap (EasyCap BC-TMS-64, EasyCap, Germany). EEG positions relative to each participant’s MRI were digitized and saved in the navigation system. EEG signals were sampled at 5 kHz (device filter DC-1250Hz), and electrode CPz served as the reference online while the ground was placed at PPO1h. EEG electrode impedances were maintained below 5 kΩ.

### Optimized Sham TMS Procedure

**Fig. 1:**
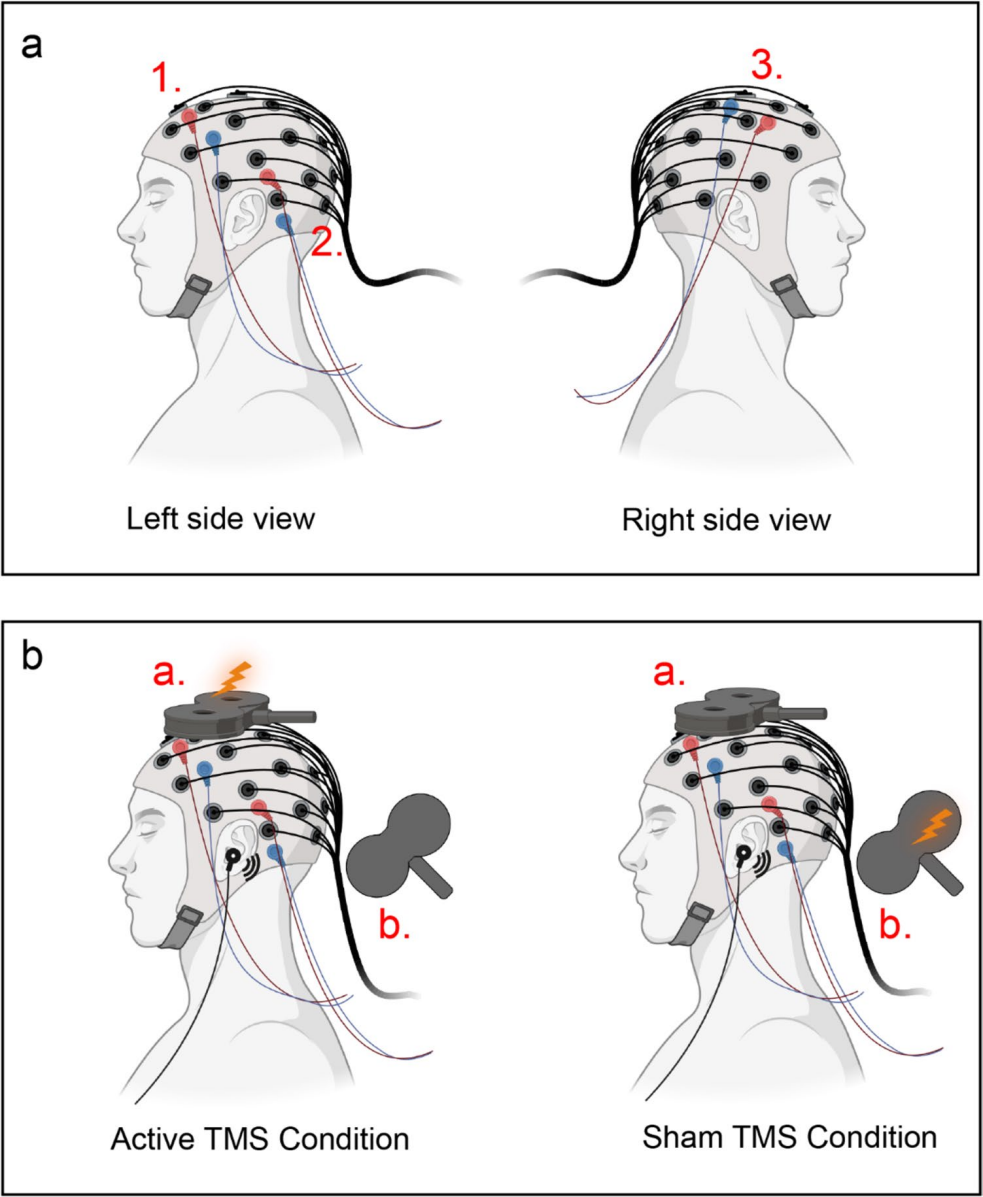
Schematic illustration of the optimized sham procedure during SMA stimulation. **a** Electrodes montage for ES. Three pairs of short-distance bipolar electrodes were attached to the EEG cap. Pair 1 (frontal: AFF1H, AFF5H) and 2 (parietal: TPP7H, TPP9H) were placed in the left hemisphere, and pair 3 (central: FFC4H, FCC4H) was in the right hemisphere. The color code used is red for the anode and blue for the cathode. **b** Experimental setup for the sham and active TMS conditions. Two identical TMS coils (**a** and **b**) were used. Coil a was positioned over the participant’s head to target SMA. Coil b was placed away from the head. Coil a was active in the active condition, while coil b was active in the sham condition. Concomitant ES (via three electrode pairs, intensity 24 mA, pulse width 200 µs), and masking noise were applied in both conditions (icons were created with BioRender.com). *ES* electric stimulation, *SMA* supplementary motor area, *TMS* transcranial magnetic stimulation (Color figure online)

The optimized sham stimulation procedure was adapted from our previous research (Gordon et al. 2021). To recreate TMS-associated somatosensory inputs for the sham TMS condition, we applied ES pulses with a stimulator (Digitimer DS7A, Digitimer Ltd.UK) through short-distance bipolar electrodes attached to the scalp. To reproduce the ’click’ sound, a TMS coil was placed away from the participant’s head, ensuring minimal magnetic-field impact on the cortex. The TMS intensity in sham was set to 1.6 times of active TMS to account for the increased distance (Gordon et al. 2021). Additionally, masking noise was played throughout the measurement to suppress the auditory perception (Massimini et al. 2005). The volume of masking noise was individually adjusted so that the ’click’ sound became barely audible but not too loud to induce discomfort. For the active TMS condition, the TMS coil positions were defined as in section “TMS–EEG setup”, and masking noise was also applied. Fig. 1 illustrates the example of targeting SMA.

To ensure comparable PEPs between the sham and active TMS conditions, we aimed to saturate the EEG response to sensory inputs. For this purpose, the scalp ES pulses were applied to both conditions with a high intensity of 24 mA (width 200 µs, maximum voltage of 400 V) through three pairs of bipolar electrodes. These electrode pairs were positioned near the three TMS targets: the first pair was in the frontal area (AFF1h and AFF5h), the second pair was in the parietal area (TPP7h, TPP9h), and the last pair was in the central area (FFC4h, FCC4h) (Fig. 1a). All pairs were fired together, and the polarity of each pair was switched after each pulse. These ES parameters were chosen according to a pilot test during which the participant reported a strong, spread but tolerable perception. For each TMS–EEG block, we recorded 150 pulses for the active TMS conditions and 50 for sham conditions, with the sham stimulation trials randomly interleaved within the active TMS conditions. Consequently, a total of 150 pulses were recorded for the sham conditions across the three TMS–EEG blocks. The interstimulus interval (ISI) was 3s (± 1s jitter; 2–4s range).

### TMS–EEG Data Preprocessing

Offline EEG analyses were performed in Matlab environment (version 2022a, MathWorks Inc.). EEGLAB (Delorme and Makeig 2004), FieldTrip (Oostenveld et al. 2011), and customized scripts were used.

Individual TMS–EEG data were preprocessed separately for each TMS target with the following steps. First, the continuous raw data were epoched around TMS pulses with a time window of − 1500 to 1500 ms, and baseline correction was done with respect to the time window of − 1000 to − 5 ms. We then concatenated the data from the sham (150 epochs) and active condition (150 epochs) before proceeding with the rest of the steps. The rationale is to ensure an equal artifact rejection procedure for both conditions and avoid bias from inconsistent data processing. A robust detrending function (3rd-order polynomial fitting) was then performed to remove the ongoing trend. To estimate the trend line based on the pre-and post-evoked EEG signal only, the time interval of − 20 to 600 ms containing the evoked potentials were excluded from the polynomial fitting (de Cheveigné and Arzounian 2018; Hernandez-Pavon et al. 2022). Subsequently, we re-segmented the data into a shorter time window (− 1000 to 1000 ms) to cut out edge artifacts. The decay artifacts were then removed with a customized fit decay function (brief description in Supplementary Materials: section 1.1). Next, TMS and ES pulse artifacts (− 4 to 17 ms) were eliminated and cubic-interpolated before resampling to 1 kHz. Channels and trials heavily contaminated by noise or artifacts were manually excluded via visual inspection. The following preprocessing steps were based on a newly proposed framework (Metsomaa et al. in submission): The ocular artifact topographies were identified using FastICA and saved for removal in a later step (Hyvarinen 1999). Next, the SSP–SOUND joint algorithm was used to estimate the signal subspace containing the TMS-related artifacts and suppress them from EEG signals. To maintain consistent spatial properties, the saved ocular topographies were also modified with the same SSP–SOUND correction matrix as the data. Data were then re-referenced to the average, and ocular artifacts were corrected using the beamforming filter with the modified ocular topographies (Hernandez-Pavon et al. 2022). In the end, the removed channels were interpolated using spherical interpolation based on the surrounding channels. We then separated data into sham and active TMS conditions. The epochs within each condition were averaged, resulting in the evoked EEG potentials. To obtain the ’cleaned’ TEPs, the evoked potential from the sham was subtracted from the active conditions (Active-Sham) (Gordon et al. 2021).

### Analysis and Statistics

#### Visual Analogue Scales VAS

The VAS scores regarding the perception of auditory and somatosensory inputs in the active TMS and sham conditions were analyzed using R software. To assess the differences in perception between active TMS and sham conditions, we compared each sensation item using the Wilcoxon signed-rank test (for two dependent conditions) for each TMS target. The difference was considered significant at p < 0.05.

#### Global Mean Field Amplitude (GMFA)

GMFA is the standard deviation of voltage values across all electrodes at a given time point. As shown below, $t$ is time, ${V}_{i}$ is the voltage at channel $i$, ${V}_{mean}$ is the mean of the voltage over all channels, and $K$ is the number of channels.

1 $$GMFA=\sqrt{\frac{{\sum }_{i}^{k}{\left({V}_{i}\left(t\right)-{V}_{mean}\left(t\right)\right)}^{2}}{K}}$$

Besides indicating global EEG activities, GMFA was also used to identify time windows of interest (TOIs). The determination of TOIs involved using a peak detection algorithm to divide the 20–300 ms post-stimulation window into shorter epochs based on the detected peaks (Rogasch et al. 2020). Specifically, the algorithm was applied to the mean GMFA of sham and active conditions, ensuring an independent data selection approach that is uncorrelated with the condition comparison (Cohen 2014). As early peaks were not identifiable in the mean GMFA, peaks (or troughs) were extracted from the mean evoked potentials of sham and active conditions, averaged across electrodes near the TMS target. This approach resulted in the identification of five TOIs for each target.

#### Test–Retest Reliability

The test-retest reliability of TMS–EEG measures was assessed via the intersession concordance correlation coefficient (CCC). CCC is a form of intraclass correlation coefficient to assess agreement between partitions. It has been used for evaluating the test-rest reliability of TEPs across repeated tests (Bertazzoli et al. 2021; Kerwin et al. 2018; Moffa et al. 2022; Schambra et al. 2015).

2 $$CCC = \frac{2{\sigma }_{12}}{{\sigma }_{1}^{2}+{\sigma }_{2}^{2}+{\left({u}_{1}-{u}_{2}\right)}^{2}}$$

where ${\sigma }_{12}$ is the covariance between two partitions, ${\sigma }_{x}$ is the variance, ${\mu }_{x}$ is the mean (Lawrence and Lin 1989). Inter-session CCCs for TMS–EEG responses were calculated pair-wisely between sessions (S1 v S2, S1 v S3, S2 v S3) in spatial (for each given time point across all electrodes) and temporal (for each given electrode across TOIs) domains. For temporal reliability, CCCs were calculated within each TOI and the whole time window. Since the estimated CCCs follow an asymptotic normal distribution, the inverse hyperbolic tangent transformation (Fisher z-transformation) was used to improve the approximation to a normal distribution (Lawrence and Lin 1989). At the group level, CCCs were averaged across participants. We expected the CCC values to fluctuate around zero if the EEG responses could not be replicated between sessions. Therefore, one-sample permutation t-tests were applied with the null hypothesis that CCCs from each time point (spatial correlation) or each electrode (temporal correlation) were no different from zero. Multiple comparisons were controlled by the t_max_ method (Blair and Karniski 1993). Averaged CCCs were transformed back to the original scale with inverse Fisher z-transformation for visualization. In addition, we employed a scale proposed by (Shrout 1998) to interpret CCC values as in previous research (Bertazzoli et al. 2021; Moffa et al. 2022): 0.00–0.10 virtually no reliability, 0.11–0.40 slight, 0.41–0.60 fair, 0.61–0.80 moderate, and 0.81–1.0 substantial reliability. Additionally, the CCCs were also examined at the individual level to explore whether the TMS-EEG responses were stable across sessions within and between individuals (Ozdemir et al. 2021). The methods and results can be found in Supplementary Materials: Sects. 1.2 and “Participant and general procedure”.

#### Comparing the Evoked EEG Responses to the Active and Sham TMS

We next investigated the difference in the EEG spatiotemporal profile between the active and sham conditions for each TMS target. The cluster-based permutation t-test was applied to compare evoked EEG potentials from both conditions across time points and electrodes (Maris and Oostenveld 2007). Since the size of later clusters could bias the detection of smaller earlier clusters, statistical analysis was performed within each TOI instead of the whole time window. The statistical significance was assessed by testing the null hypothesis that evoked EEG responses were exchangeable between active and sham conditions (cluster threshold: p < 0.05 dependent t-test, alpha < 0.05 two-tailed; randomization = 2000), and the critical alpha level was corrected with Bonferroni by the number of TOIs.

To better visualize cortical activation, we projected the scalp EEG signals from the active and sham conditions to source space using l2-minimum-norm estimation (MNE). For the forward solution, each participant’s T1 image was processed through a pipeline involving the Fieldtrip, FreeSurfer software (Fischl 2012), HCP-workbench, and SPM toolboxes jointly with custom-made scripts. The source model was built on a triangulated cortical mesh with 15,684 vertices. The resulting cortical meshes were surface-registered to a common spherical template, which enables direct comparison of source locations with the same index across participants. A 3-D compartment volume conductor model was created individually with the Boundary Element Method (Stenroos and Nummenmaa 2016). Three meshes were constructed to define the following compartments: inner skull, outer skull, and scalp. The conductivity inside each boundary surface was set to 0.33 S/m, 0.0041 S/m, and 0.33 S/m, respectively. EEG electrode positions were aligned with the generated scalp surface. For the inverse estimation, the cortical distributed sources were assumed to have fixed orientations (perpendicular to the gray-white matter surface). The source covariance matrix was assumed such that nearby sources were more correlated than distant sources (according to Gaussian as a function of distance), and Tikhonov regularization was used for numerical stabilization of the matrix inverse (more details in Supplementary Materials: sect. 1.3). The reconstructed current source density for evoked EEG responses was z-score normalized with respect to a pre-stimuli time window (− 600 to − 100 ms). At group level, normalized current density was averaged across participants, and the spatiotemporal patterns were displayed on the common cortical template.

## Results

### VAS: Active TMS Condition vs. Sham TMS Condition

**Fig. 2:**
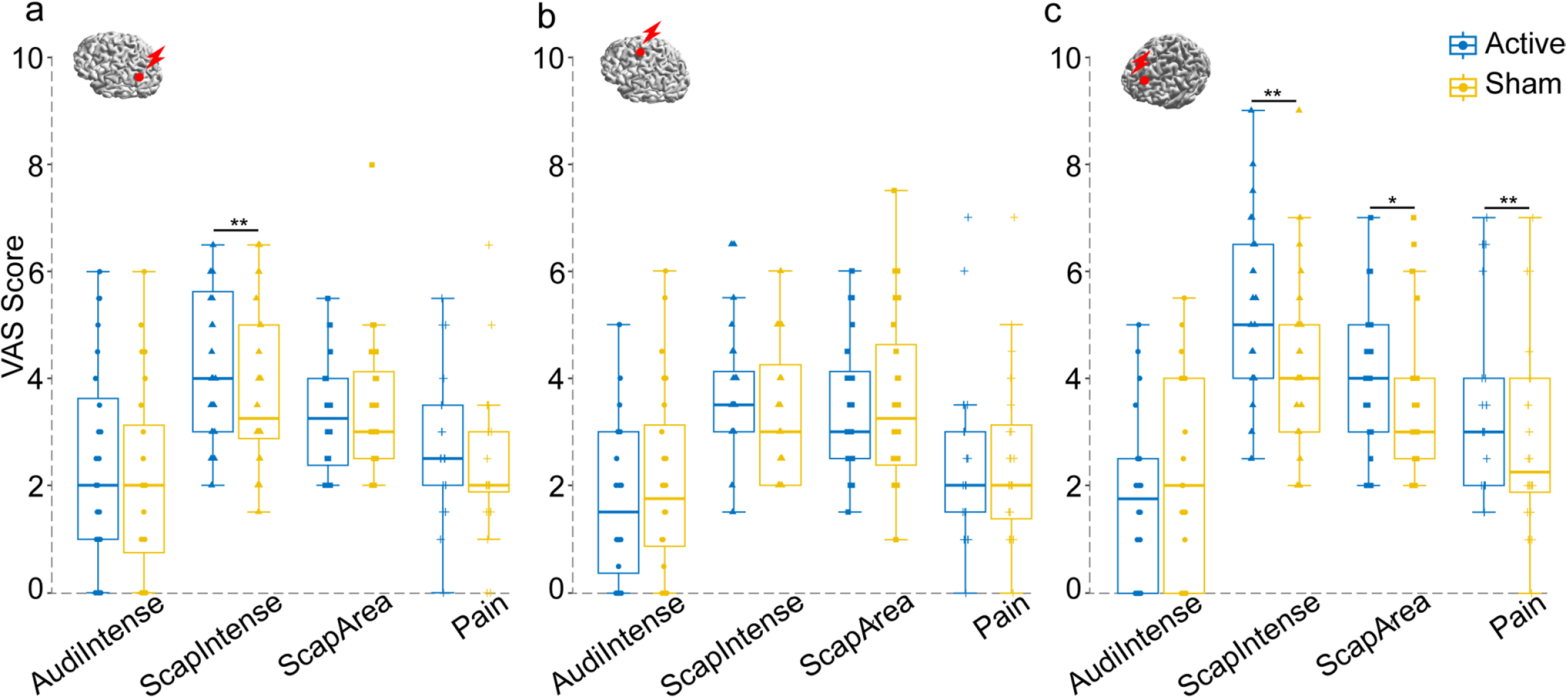
Comparison of perceptions in active TMS vs. sham TMS conditions. Box plots show the VAS scores for the four perception items: auditory intensity (AudiIntense), scalp intensity (ScapIntense), scalp area size (ScapArea), and pain or discomfort (Pain) following AG (**a**), SMA (**b**), and mPFC (**c**) stimulations, *p < 0.05; ** p < 0.001.* AG* angular gyrus, *mPFC* medial prefrontal cortex, *SMA* supplementary motor area, *TMS* transcranial magnetic stimulation, *VAS* visual analogue scale

The differences in VAS scores between active and sham TMS conditions are shown in Fig. 2. For AG stimulation, a significant difference was present only in the intensity of scalp between the active TMS and the sham condition (auditory intensity: p = 0.393; scalp intensity: p = 0.005; scalp area size: p = 0.659; pain: p = 0.096;). However, there were no significant differences in perception between the two conditions when SMA was stimulated (auditory intensity: p = 0.111; scalp intensity: p = 0.086; scalp area size: p = 0.778; pain: p = 0.904;). For mPFC stimulation, the perception of the scalp intensity, the scalp area size, and the discomfort significantly differed between the two conditions, but not for the auditory intensity (auditory intensity: p = 0.075; scalp intensity: p = 0.001; scalp area size: p = 0.026; pain: p = 0.008).

### Test–Retest Reliability of TMS–EEG Responses

**Fig. 3:**
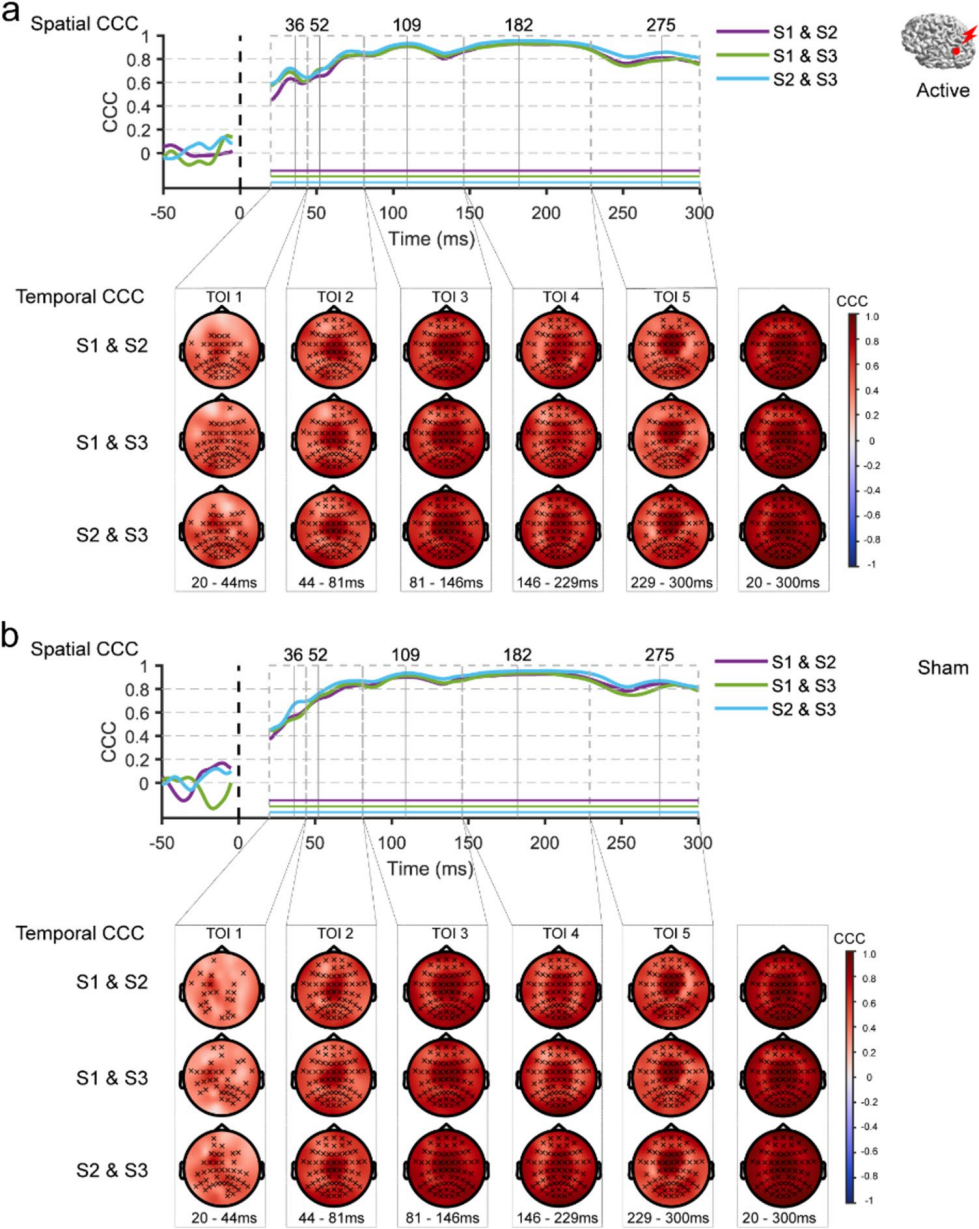
Test–retest reliability of the evoked EEG potentials by active TMS (**a**) and sham TMS (**b**) of AG. The red dot on a template brain indicates the cortical target. The upper panel shows the spatial inter-session CCCs, with traces (purple, green, and blue) representing the group averages of CCCs for each pair of sessions. Horizontal lines indicate time points where CCCs significantly differ from zero. The lower panel displays topography of the temporal CCCs within each TOI, x indicating electrodes with CCCs significantly different from zero. *AG* angular gyrus, *CCC* concordance correlation coefficient, *S1*, *S2*, *S3* sessions 1–3, *TMS* transcranial magnetic stimulation, *TOI* time window of interest (Color figure online)

We first assessed how reliable TMS–EEG responses were between repeated sessions. To this end, inter-session CCCs for EEG responses from the sham and the active TMS were calculated in spatial and temporal domains. For all TMS targets, the values of spatial CCCs from the active and sham conditions reached a high level (CCC > 0.8) from approximately 90 ms onwards. Likewise, significantly high temporal CCCs were observed at most electrodes after the earliest TOI. Figure 3 illustrates the spatiotemporal reliability pattern of the EEG responses elicited by active TMS when targeting AG. Similar patterns were observed for the sham and active TMS conditions at mPFC and SMA (Figs. S1, S2 in Supplementary Materials).

**Fig. 4:**
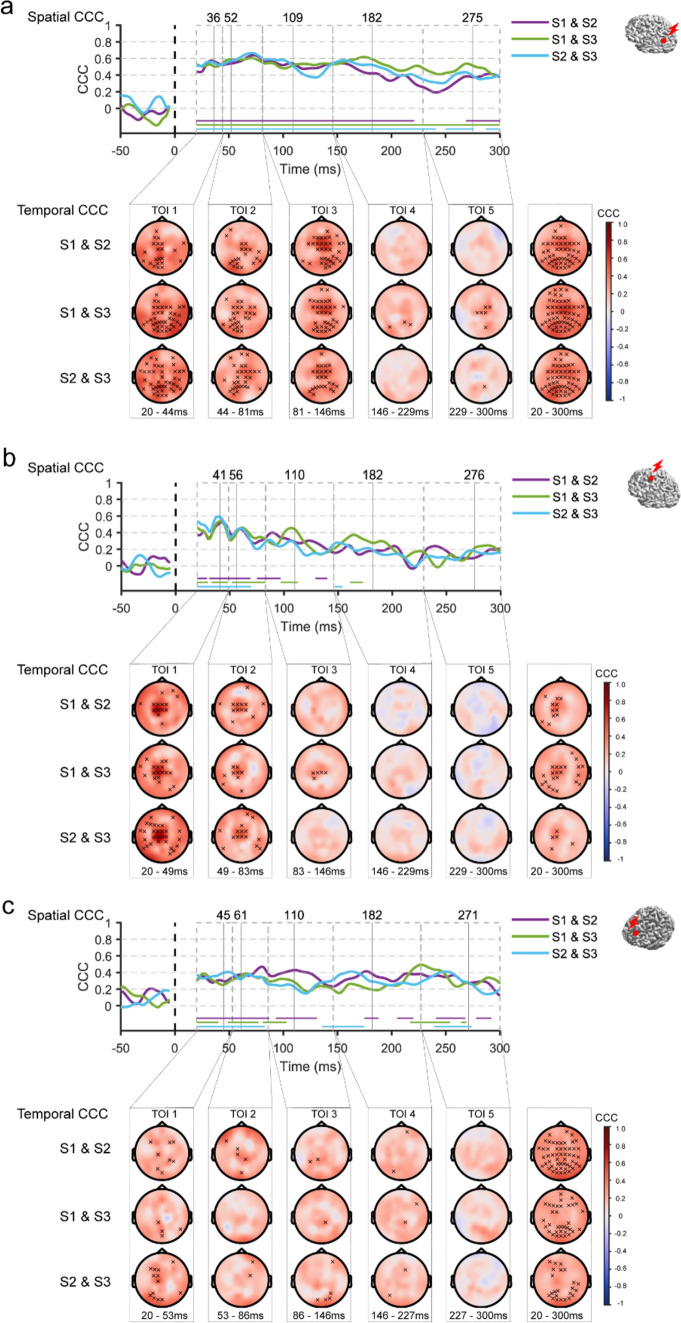
Test–retest reliability of the’cleaned’ TEPs (Active-Sham) following AG (**a**), SMA (**b**) and mPFC (**c**) stimulations. The red dot on a template brain indicates the cortical target. The upper panel shows the spatial inter-session CCCs, with traces (purple, green, and blue) representing the group averages of CCCs for each pair of sessions. Horizontal lines indicate time points where CCCs significantly differ from zero. The lower panel displays topography of the temporal CCCs within each TOI, x indicating electrodes with CCCs significantly different from zero. *AG* angular gyrus, *CCC* concordance correlation coefficient, *mPFC* medial prefrontal cortex, *S1*, *S2*, *S3* sessions 1–3, *SMA* supplementary motor area, *TMS* transcranial magnetic stimulation, *TOI* time window of interest (Color figure online)

Given the similar results observed between the active and sham TMS conditions, it is likely that PEPs explain the high spatiotemporal CCCs between sessions in the active TMS condition. To investigate whether EEG responses remained highly reliable after the removal of PEPs, we further assessed the CCCs for the ’cleaned’ TEPs (Active-Sham) in spatial and temporal domains between sessions. TMS of AG resulted in significant spatial CCCs over the first 200 ms (Fig. 4a). Specifically, a fair to moderate CCC (0.4 < CCCs < 0.67) was observed until approximately 190 ms, with a slight decrease after 100 ms. Many electrodes displayed significant temporal CCCs up to 150 ms after the TMS pulse. For SMA, spatial CCCs remained consistently significant until around 80 ms after the TMS pulse (0.2 < CCCs < 0.6), but the values dropped considerably thereafter. Temporal CCCs were highly significant within the first 80 ms in electrodes in the central area, corresponding to the targeted area (Fig. 4b). For mPFC, spatial CCCs were significant until approximately 80 ms after the TMS pulse (0.2 < CCCs < 0.47). However, low temporal CCCs were observed across the scalp, with few electrodes reaching significance between sessions (Fig. 4c). These results showed that inter-session CCC values for late EEG responses decreased after the removal of PEPs. Notably, significant spatial CCCs for early ’cleaned’ TEPs were found for each target, while the strength of temporal CCCs varied.

### Evoked EEG Responses to Active TMS vs. Sham TMS

We next assessed differences in the evoked EEG responses between the active and sham TMS conditions. The high test-retest reliability (as shown in Fig. 3 and Figs. S1, S2) indicated stable and consistent evoked EEG responses from both active and sham TMS conditions between sessions, so we combined the stimulation trials across the three sessions for each condition, aiming to maximize the signal-to noise ratio (SNR) of the evoked responses to both active and sham conditions. For each TMS site, the evoked potentials by the active and sham TMS conditions were compared using the cluster-based permutation t-test.

**Fig. 5:**
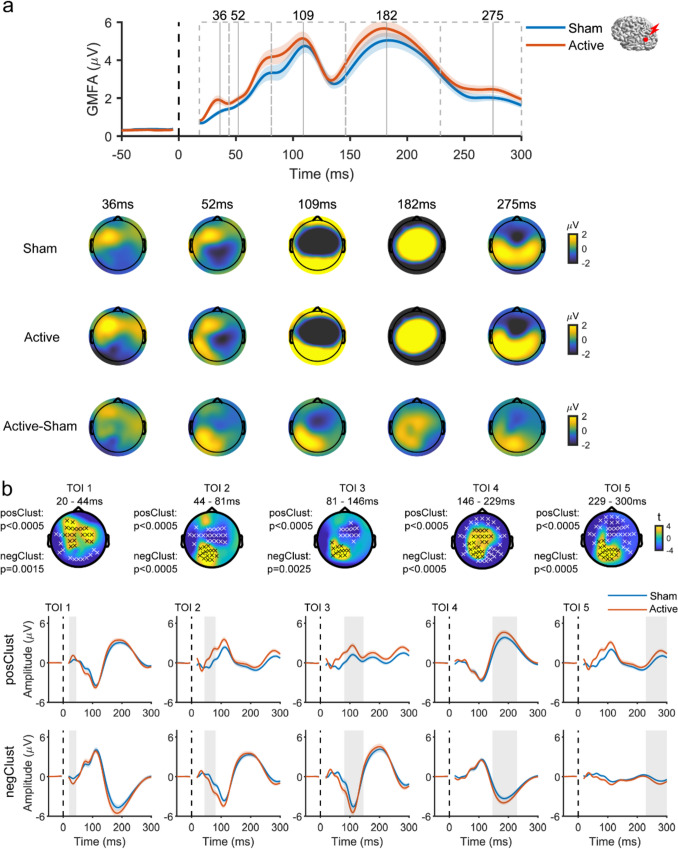
Comparison of the evoked EEG potentials by active TMS vs. sham TMS of AG **a** Above, GMFA of the evoked EEG potentials. The solid lines (blue: sham, red: active) represent the group averages, and shaded areas indicate the standard error. Dashed boxes represent TOIs with vertical lines indicating the timing of peaks. Below, topographies show the spatial distribution of the evoked EEG potentials at peak latencies in three conditions: sham (top row), active (middle row), and ‘cleaned’ (active-sham; bottom row). **b** Results of cluster-based t-test. Topographies show t-statistic maps within each TOI. Above, electrodes that contribute to significant positive and negative clusters are highlighted with black x and white x, respectively. The corresponding p values are displayed to the left. Below, evoked EEG potentials were averaged across electrodes comprising the significant clusters (top: positive, bottom: negative). The solid lines (blue: sham, red: active) represent the group averages, and the shaded areas are the standard error. Shaded grey columns correspond to the TOIs.* AG* angular gyrus, *GMFA* global mean field amplitude, *TMS* transcranial magnetic stimulation, *TOI* time window of interest (Color figure online)

**Fig. 6:**
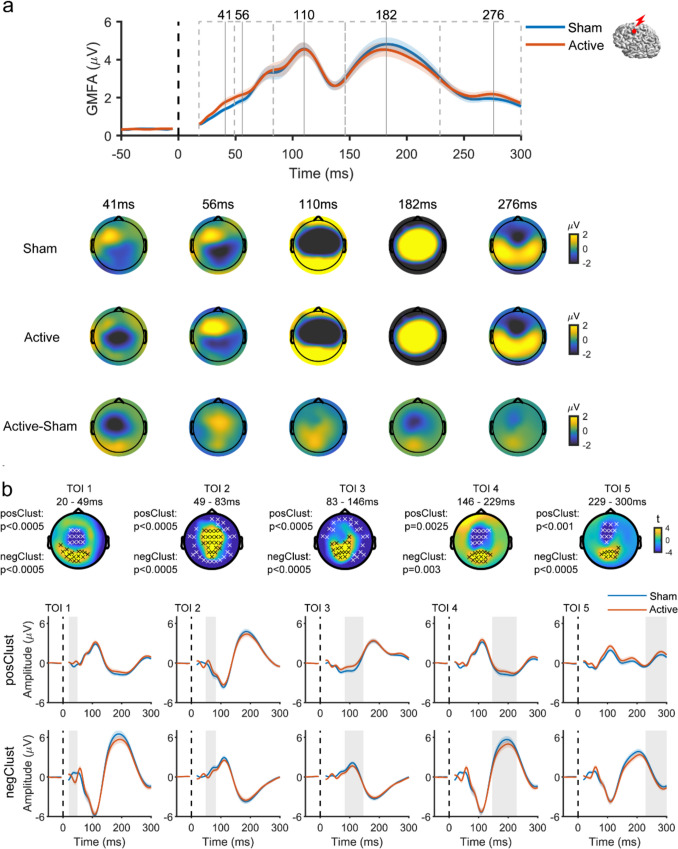
Comparison of the evoked EEG potentials by active TMS vs. sham TMS of SMA **a** Above, GMFA of the evoked EEG potentials. The solid lines (blue: sham, red: active) represent the group averages, and shaded areas indicate the standard error. Dashed boxes represent TOIs with vertical lines indicating the timing of peaks. Below, topographies show the spatial distribution of the evoked EEG potentials at peak latencies in three conditions: sham (top row), active (middle row), and ‘cleaned’ (active-sham; bottom row). **b** Results of cluster-based t-test. Topographies show t-statistic maps within each TOI. Electrodes that contribute to significant positive and negative clusters are highlighted with black x and white x, respectively. The corresponding p values are displayed to the left. Below, evoked EEG potentials were averaged across electrodes comprising the significant clusters (top: positive, bottom: negative). The solid lines (blue: sham, red: active) represent the group averages, and the shaded areas are the standard error. Shaded grey columns correspond to the TOIs.* GMFA* global mean field amplitude, *SMA* supplementary motor area, *TMS* transcranial magnetic stimulation, *TOI* time window of interest (Color figure online)

**Fig. 7:**
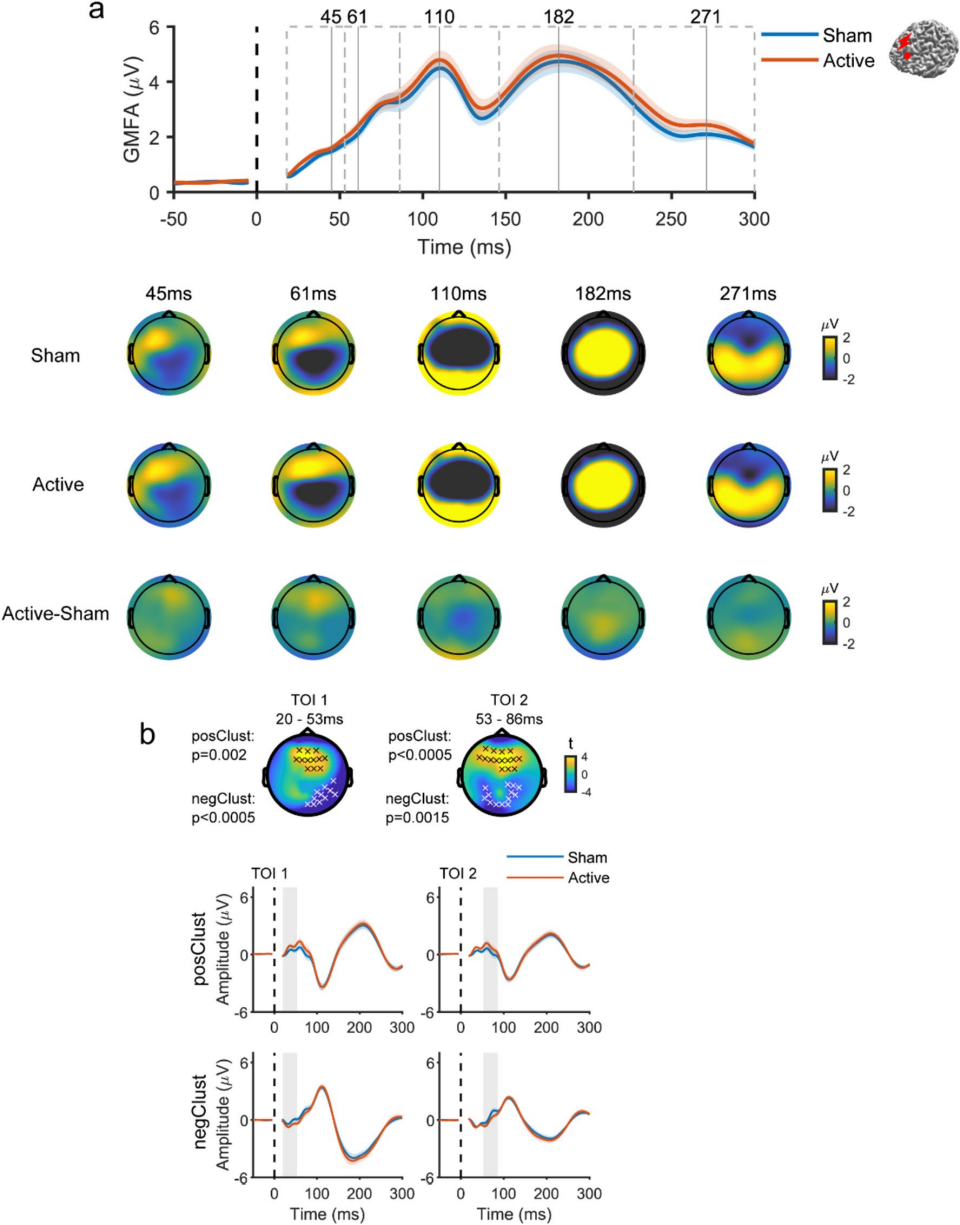
Comparison of the evoked EEG potentials by active TMS vs. sham TMS of mPFC **a** Above, GMFA of the evoked EEG potentials. The solid lines (blue: sham, red: active) represent the group averages, and shaded areas indicate the standard error. Dashed boxes represent TOIs with vertical lines indicating the timing of peaks. Below, topographies show the spatial distribution of the evoked EEG potentials at peak latencies in three conditions: sham (top row), active (middle row), and ‘cleaned’ (active-sham; bottom row). **b**. Results of cluster-based t-test. Topographies show t-statistic maps within each TOI. Electrodes that contribute to significant positive and negative clusters are highlighted with black x and white x, respectively. The corresponding p values are displayed to the left. Below, evoked EEG potentials were averaged across electrodes comprising the significant clusters (top: positive, bottom: negative). The solid lines (blue: sham, red: active) represent the group averages, and the shaded areas are the standard error. Shaded grey columns correspond to the TOIs.* GMFA* global mean field amplitude, *mPFC* medial prefrontal gyrus, *TMS* transcranial magnetic stimulation, *TOI* time window of interest (Color figure online)

Despite the similarity of responses from both conditions, cluster analysis revealed significant differences in evoked EEG responses between the active and sham TMS conditions when applying TMS to the AG. Specifically, TMS of AG led to a higher positive amplitude deflection in frontal electrodes within the initial 40 ms, followed by a higher amplitude positive deflection at the left parietal area (the targeted region) up to and beyond 300 ms. In addition, increased response amplitude was detected in the central area at around 200 ms (Fig. 5). TMS of SMA resulted in a negative deflection in the central area (the targeted region) within the first 50 ms, significantly differing from sham. The deflection transitioned into positive with significantly larger amplitude until 80 ms, then again became negative from 150 ms until and beyond 300 ms (Fig. 6). TMS to the mPFC resulted in a significantly higher amplitude positive deflection around the prefrontal area (the targeted region) within the first 80 ms when compared to the response from sham TMS (Fig. 7). These results showed that spatially distinguishable differences between the EEG responses in the active and sham TMS conditions could be detected for each TMS target.

**Fig. 8:**
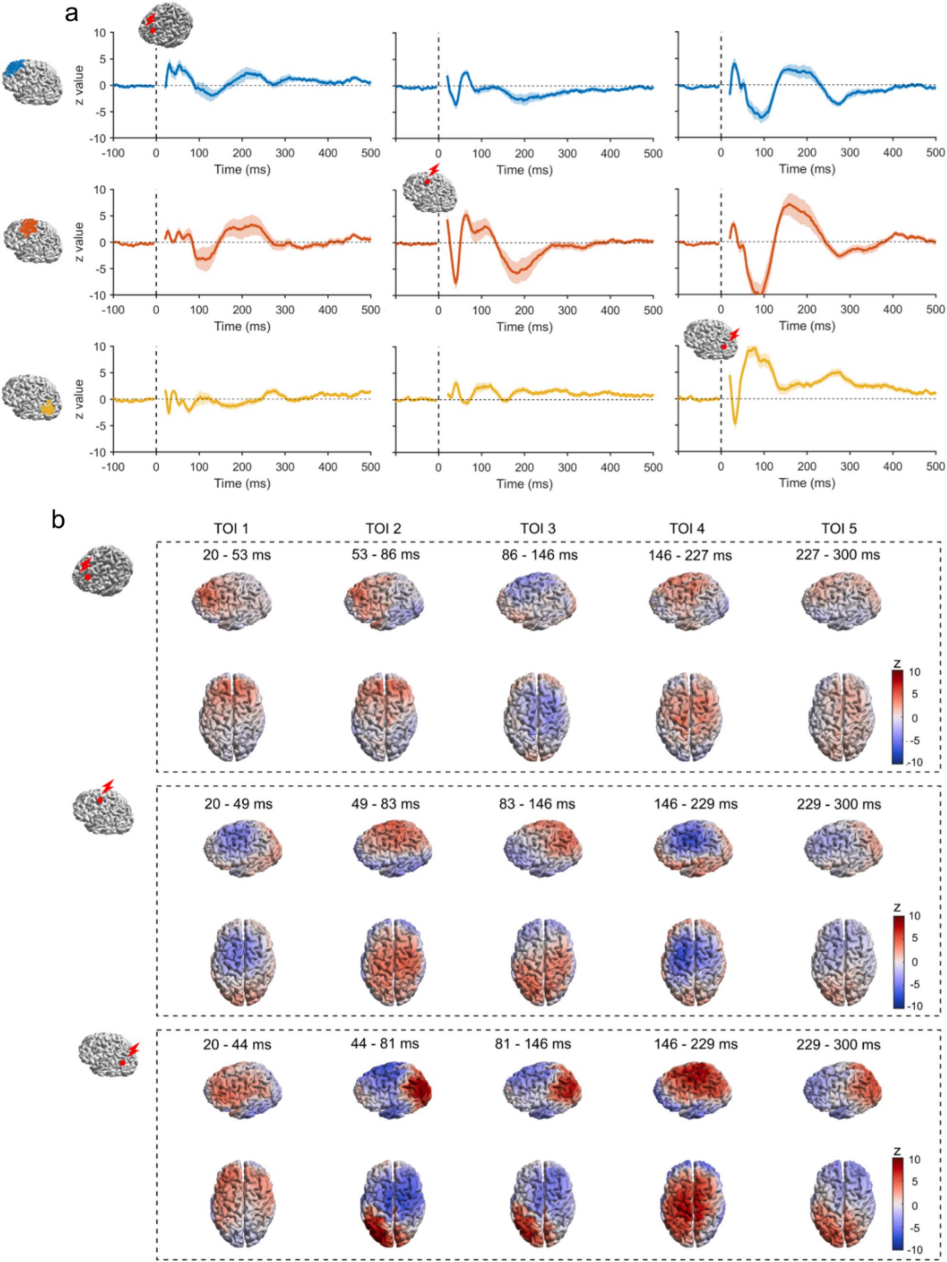
Source activation pattern. **a** Temporal dynamics of source activation following mPFC (left column), SMA (middle column), or AG (right column) stimulations. The red dot on a template brain indicates the cortical target. The time series of normalized current density were averaged within the ROIs: mPFC (blue; top row), SMA (red; middle row), and AG (yellow; bottom row). The solid trace is the group average, and the shaded areas are the standard error. Plots on the diagonal are the effects of TMS on local cortical regions. Plots off the diagonal are the effects of TMS on distal cortical regions. **b** Spatial propagation of source activation following mPFC (top row), SMA (middle row), or AG (bottom row) stimulations. The normalized current density was averaged within the TOIs, and the spatial distribution of the group average is visualized from the left side and top views.* AG* angular gyrus, *mPFC* medial prefrontal gyrus, *ROI* region of interest, *SMA* supplementary motor area, *TMS* transcranial magnetic stimulation, *TOI* time window of interest (Color figure online)

To better visualize cortical activation following TMS at different targets, we applied source estimations to the evoked EEG responses from both sham and active TMS conditions. Sham responses were subtracted from the active TMS response. Three cortical regions of interest (ROIs) were chosen, corresponding to mPFC, SMA and AG (Fig. 8). The time series of local source activation was obtained by averaging the dipole activities within each ROI. Rapidly changing deflections were observed shortly after the TMS pulse for each target and subsided to baseline at around 300 ms. The spatial distribution illustrates the propagation of the TMS-evoked EEG response as time evolved. Instead of remaining localized to the targeted area, we noticed that the activation due to TMS propagated to distal regions. The evolved propagation became more apparent when inspecting the animations (see Animations 1-3 in Supplementary Materials for mPFC, SMA and AG, respectively). Reciprocal propagation of activation between prefrontal and parietal regions was observed when mPFC and AG were stimulated. In contrast, when stimulating SMA, the activation was more confined to the central and left sensorimotor cortex.

## Discussion

We assessed the EEG responses resulting from TMS of three brain regions, the mPFC, AG, and SMA, focusing on their differences from EEG responses to multisensory inputs and their test-retest reliability. It is essential to distinguish the EEG responses arising from direct cortical activation and those resulting from multisensory co-stimulation. Failure to do so could leads to erroneously interpreting PEPs as ’true’ TEPs (Biabani et al. 2019; Conde et al. 2019). To this aim, we used a recently developed optimized sham procedure that can consistently remove the entirety of the overlapping PEPs (Gordon et al. 2021). Our study yielded several findings. First, significant differences in the EEG responses between the active and the sham TMS conditions were revealed for all TMS targets, mainly in the first 90 ms after the TMS pulse. Moreover, specific spatial and temporal characteristics of these EEG responses varied depending on each target. Lastly, the test-retest reliability of late EEG responses considerably decreased after removing PEPs, in particular at latencies > 80 − 100 ms after the TMS pulse, and the reliability of early responses < 80 ms was variable across the targeted areas.

### Separation of TEPs from PEPs

We found significant differences in EEG responses between the active and sham TMS conditions within the first 90 ms for all targets. Notably, these differences were centered around the respective targeted regions. Source estimation further demonstrated that the activation of cortical regions near the targeted area most likely accounted for the site-specific difference. Unlike the early responses (< 90 ms), which are known to be relatively unaffected by PEPs (Belardinelli et al. 2019; Conde et al. 2019; Gordon et al. 2021; Rogasch et al. 2020), the late TEPs can be heavily contaminated by components such as N100 and P200 (Biabani et al. 2019; Conde et al. 2019). N100 and P200 are commonly identified PEP components and various brain areas are thought to be involved, including primary and secondary somatosensory cortices, superior temporal cortex, insula, posterior and anterior cingulate cortices, and frontal cortex (Mouraux and Iannetti 2009). Thus, N100 and P200 typically exhibit large amplitudes with a broad frontocentral scalp distribution, whereas the ’true’ TEPs have comparatively smaller amplitudes and greater topographical specificities, which can be overshadowed by the presence of PEPs. Nevertheless, when stimulating SMA, we identified a positive component of ’cleaned’ TEPs at around 100 ms and a negative one at 200 ms in the central area (i.e., with opposite polarity to N100-P200). Moreover, the source estimation suggests these components are more evident in the central and left sensorimotor cortex (as shown Fig. 8b: middle row), which differs from the widespread PEPs with amplitudes usually greater in the hemisphere contralateral to stimulation (Hashimoto 1988). When stimulating AG, a negative component of ’cleaned’ TEPs at around 100 ms and a positive at 200 ms in the central area were detected, which can also be inspected at the source level (Fig. 8b: top row). However, these responses more likely correspond to residual PEPs, in which the sensory inputs from the sham did not match those from the active TMS. The significant difference in VAS scores (the intensity of scalp sensation) between the sham and the active TMS condition may support this explanation (Fig. 2a). Interestingly, for mPFC stimulation, despite evident differences in perceptions between conditions (Fig. 2c), no significant components were detected in the late latencies of ‘cleaned’ TEPs (Fig. 7), indicating that PEPs from both conditions were considerably equivalent.

Besides the confinement of ’cleaned’ TEPs to each targeted area (Farzan and Bortoletto 2022), our findings from stimulating three distinct areas provide further evidence of successful measures of ’true’ TEPs. First, although the same sham TMS procedure was applied to all three active TMS conditions, comparing responses from each active TMS target to the sham responses revealed significant differences with specific spatiotemporal patterns that varied depending on each target. This is highly likely the result of direct cortical activation of each targeted area rather than unspecific cortical responses to sensory inputs. Moreover, these site-specific EEG responses suggest that different neuronal populations were recruited, adding to the evidence that TEPs reflect the effects of TMS on local cortical circuitry (Li et al. 2017; Romero et al. 2019; Rosanova et al. 2009). Lastly, responses at the source level provided a more accurate visualization of the propagation of the TMS effects. Depending on the target, the propagation of activation was either between the frontal and parietal cortex (TMS of mPFC and AG) or more confined to the central and left somatosensory cortex (TMS of SMA) (Fig. 8, and Animations 1-3 in Supplementary Materials). The specific propagation patterns suggest that different brain regions, structurally and functionally connected to the targeted areas, were engaged during the time of stimulation. Our findings provide additional evidence that the TEPs have the potential to index network-level dynamics (Bortoletto et al. 2015; Ozdemir et al. 2020).

### Reliability of TEPs Following PEPs Removal

Recently, several studies assessed the test-retest reliability of TEPs resulting from the left dorsolateral prefrontal cortex stimulation using the inter-session CCC, a metric that is better suited to determine the agreement of measures between repeated tests (Bertazzoli et al. 2021; Kerwin et al. 2018; Moffa et al. 2022). These studies consistently reported that late TEP components had better reliability between sessions than the early ones, with CCC values reaching the moderate and substantial range. Though auditory masking and foam padding were implemented in these studies, they have been considered inadequate in dealing with PEP contamination (Conde et al. 2019; Ross et al. 2022). Hence, the high reliability after 100 ms is likely attributed to residual PEPs such as the N100-P200 components. Due to their greater amplitude and broader spatial distribution, N100-P200 tends to be more stable and reliable. Accordingly, in our study, the inter-session CCCs for evoked EEG responses in the active TMS conditions reached a substantial level from around 90 ms onwards, i.e., during the presence of PEPs. However, after removing PEPs, the CCC values decreased markedly for the ‘cleaned’ TEPs for SMA and mPFC. In contrast, the comparatively higher CCC values beyond 100 ms following TMS of AG are likely the results of residual PEPs.

Regarding early responses, we found that ‘cleaned’ TEPs generally showed significant spatial reliability within the first 90 ms. When stimulating AG and SMA, electrodes near TMS targets tended to show better temporal reliability. This is consistent with the spatiotemporal profile of ’true’ TEPs, and it further suggests that these early responses are physiologically meaningful rather than the result of PEPs or unrelated cortical activity. However, lower temporal reliability with few electrodes reaching significance was observed following TMS of mPFC. Likewise, two recent studies reported either slight to fair (P20, P50) (Bertazzoli et al. 2021) or little test-retest reliability (N40, P20) (Moffa et al. 2022) of TEPs resulting from stimulating dorsolateral prefrontal cortex as measured by inter-session CCCs. The inter-individual variability, as suggested by the low between-participants CCC values (Fig. S3 and Table S1), could partially explain the poor test-retest reliability of TEPs in response to mPFC stimulation. The higher TMS intensity (120% RMT) used in our study might be another reason. It was intended to increase cortical response based on the observation of relatively smaller TEPs when stimulating the prefrontal and parietal cortex with an intensity of 100% RMT (Rogasch et al. 2020). Nevertheless, this may have contributed to low SNR of TEPs due to amplified noise at the same time, such as muscle artifacts in the frontal regions. In this regard, TMS-induced E-field estimates may help determine TMS intensity, but the desired E-field (strength, orientation) for neuronal activation in mPFC is still unknown (Hernandez-Pavon et al. 2023; Janssen et al. 2014). In addition, the online evaluation of TMS-EEG responses has also been recommended for searching optimal TMS parameters (intensity, coil orientation and location) (Casarotto et al. 2022). Yet, it may not be advantageous for targeting frontotemporal regions where the blinks and cranial muscle twitches could overwhelm the real-time readout. Future studies should explore strategies that can optimize the SNR to enhance the reliability of TEPs in frontal regions (Parmigiani et al. 2022).

### Limitations

One methodological concern about the optimized sham design is the potential modulation effect of concomitant ES on the ’true’ TEPs. The strong sensory inputs induced by the high-intensity ES might directly change the cortical excitability or indirectly alter brain states through attention and saliency-related processes. Furthermore, this hypothesis would imply that the PEPs and the ’true’ TEPs may not be independent and linearly separable phenomena. Thus, simply subtracting evoked EEG responses by sham from active TMS conditions or statistical comparisons between them may be physiologically inappropriate. Nonetheless, it is a common concern in the TMS–EEG field that the ’true’ TEPs might be altered by unwanted sources. For instance, the inevitable multisensory co-stimulations (e.g., facial and trigeminal nerve activation) might intrinsically modify cortical excitability and brain states at the time of stimulation (Hernandez-Pavon et al. 2023; Mizukami et al. 2019; Pellegrino et al. 2022) and lead to changes of the brain’s responsiveness to TMS. However, our recent study targeting left M1 using such an optimized sham design showed that, at least at the macroscopic level of EEG, there is no evidence supporting the presence of any interaction between true TEPs and PEPs (Gordon et al. 2023). In light of this, it appears justified to employ the linear assumption as used in some TMS–EEG data cleaning methods (e.g., ICA (Biabani et al. 2019) and SSP–SIR (Mutanen et al. 2016)) and in the subtraction of sham from active TMS.

Another limitation is the residual impact of PEPs on late EEG responses following the TMS of AG, which implies that the PEPs elicited by the sham and active TMS were not equally matched. The mismatch may be due to the engaged cranial nerves and fibers for generating SEPs needing even higher ES to saturate. Because a fixed ES intensity was used for all conditions, it may underestimate the intensity to reach saturation when stimulating AG. To address this, an online titration method could be used, which involves recording simultaneous EEG with gradually increasing ES intensities until a steady PEP is reached (Gordon et al. 2021). This may help to tailor ES parameters for saturation purposes. In addition, the residual auditory perception of TMS pulses was reported in all conditions despite the use of masking noise (Fig. 2). Over-ear protection might help, but the complete suppression of the TMS ’click’ sound may not be achievable when applying high-intensity stimulation, which could be necessary for certain cortical targets and individuals with high RMT (Conde et al. 2019; Ross et al. 2022). Nevertheless, our experimental design led to a matched auditory perception in sham and active conditions at the group level, and the auditory effects might be mathematically removed with the comparison between conditions. In summary, though the optimized sham procedure helps disentangle the sensory effects on TEPs, future studies should assess multisensory contributions as a function of the target region and calibrate the sham TMS design accordingly.

Lastly, implementing concomitant ES introduces new sources of noise and exacerbates decay artifacts. Combining the stimulation trials across three sessions allowed us to increase the number of trials per condition and enhance the SNR of the evoked EEG responses, which enabled more robust statistical comparisons between the active and sham TMS conditions. However, a potential limitation of pooling data from multiple sessions is the assumption of stable and reproducible EEG responses over time, which need to be carefully considered and assessed as indicated by the high test-retest reliability in our study (Fig. 3 and Figs. S1, S2). A more sensible way is to obtain a higher SNR during single-session TMS–EEG recording, possibly by reducing the intensity of ES or recording a higher number of trials, but this might cause lengthy experiments with limited benefits.

## Conclusions

In the present study, we assessed the EEG responses resulting from TMS of three brain regions, the AG, SMA and mPFC, using an optimized sham design. We conclude that EEG responses to the direct cortical activation by TMS can be revealed within the first 90 ms for all TMS targets. Moreover, the spatial and temporal characteristics of the revealed EEG responses are specific to each target and most likely reflect the effects of TMS on local cortical circuits and networks. Lastly, the test-retest reliability of late TMS–EEG responses may be considerably affected by the presence of PEPs, and the reliability of early responses varies depending on the TMS target.

### Supplementary Information

Below is the link to the electronic supplementary material.

Supplementary file1 (DOCX 2553 KB)

Supplementary file2 (MP4 487 KB)

Supplementary file3 (MP4 537 KB)

Supplementary file4 (MP4 638 KB)

## Data Availability

The preprocessed tms-eeg data, individual head models, lead field matrices and VAS scores that support the findings of the manuscript are deposited in the repository Zenodo: https://zenodo.org/records/10004794, and the access could be granted under conditions, i.e., EULA (End User License Agreement) signed by the user. The code used is available in GitHub: https://github.com/Song-Yufei/tms-eeg-using-optimized-sham-outside-M1.
