# Supplementary material for: Evoked EEG Responses to TMS Targeting Regions Outside the Primary Motor Cortex and Their Test–Retest Reliability
Source: Brain Topogr. 2023 Nov 23;37(1):19–36. doi: 10.1007/s10548-023-01018-y (PMC10771591; doi:10.1007/s10548-023-01018-y)
Supplement: Supplementary file 1 — Supplementary file1 (DOCX 2553 KB) [file 10548_2023_1018_MOESM1_ESM.docx]

Supplementary Material

**Evoked EEG responses to TMS targeting regions outside the primary motor cortex and their test-retest reliability**

Yufei Song^1,2^, Pedro C. Gordon^1,2^, Johanna Metsomaa^1,2,3^, Maryam Rostami^4^, Paolo Belardinelli^1,2,5^, Ulf Ziemann^1,2*^

^1^Department of Neurology & Stroke, University of Tübingen, Germany

^2^Hertie Institute for Clinical Brain Research, University of Tübingen, Germany

^3^Department of Neuroscience and Biomedical Engineering, Aalto University School of Science, Finland

^4^Faculty of Electrical and Computer Engineering, University of Tehran, Iran

^5^CIMeC, Center for Mind/Brain Sciences, University of Trento, Italy

^*^Corresponding Author:

Prof. Dr. Ulf Ziemann

Department of Neurology & Stroke

Hoppe-Seyler-Straße 3, 72076 Tübingen, Germany

Email: [ulf.ziemann@uni-tuebingen.de](mailto:ulf.ziemann@uni-tuebingen.de)

## 1 Supplementary method

### 1.1 Decay artifact removal

The decay artifact was addressed using a first-degree exponential function (a+b∗exp(c∗x) applied to the time series of each epoch and channel. Here, x represented time in milliseconds, ranging from 14 to 1000 ms, and a,b, and c were the associated parameters. The fit solution was optimized using the weighted least squares method, with greater weight assigned to the initial samples after the pulse artifact to prioritize error minimization and improve accurate estimation. This weighting strategy was based on the observation that the initial samples were more likely affected by the decay artifact. The decay-corrected data were obtained by subtracting the fitted exponential model from each time series. Finally, a detrending step was applied to the epochs to minimize sudden signal drops or jumps, ensuring the signal centered around zero as much as possible.

### 1.2 Test-retest reliability at the individual level

For each cortical target, we calculated the evoked EEG potential matrix for every participant in each session (i.e., 24x3=72 matrices). Each matrix has 64 rows, one for each channel, and 280 columns, one for each time point, from 20 - 300ms post-stimulation. We then flattened the matrix into a vector and calculated the inter-session CCC between each pair of sessions using the formula (2). The results were structured into a 72 x 72 symmetric matrix (left matrix in Fig. S3a), where the rows and columns indexed 24 participants in 3 sessions. Thus, each entry in the matrix contained the CCC value calculated between a specific combination of sessions and participants. We then grouped the entries by participants, excluded the diagonal entries (self-correlation), and averaged the CCC values within every block. This produced a new 24 x 24 symmetric matrix (right matrix in Fig. S3a) where diagonal entries showed the within-participant CCCs, while off-diagonal entries indicated the between-participant CCCs.

### 1.3 Inverse estimation

We used minimum-norm estimation (MNE) to obtain source amplitudes $J$ as

$$J=\Lambda L^{T}\left( L{\Lambda L}^{T}+\Sigma\right)^{-1}Y$$

(S. 1)

where $L$ is the lead-field matrix, and $\Lambda$ and $\Sigma$ are the source and noise covariance matrices, respectively. *Y* is the EEG signal after preprocessing. As noise covariance matrix, we used the identity matrix $\Sigma=\delta I$, which means that noise is uncorrelated across sensors with constant variances, and the regularization parameter $\delta$ corresponds to the noise-to-signal ratio (inverse of the signal-to-ratio) of EEG signal. As a regularization parameter, we used 0.01. The lead-field matrix here was computed for fixed orientations of source dipoles, which means that each dipole position corresponds to one element in $J$, and one respective column in $L$. The source covariance matrix is often chosen as a scaled identity matrix, similar to the noise covariance matrix above, corresponding to independently activating sources across the cortical surface. Here, we assumed that the source amplitudes are likely to be correlated if they are near each other, and the correlation between two sources $i$ and $j$ decreases as a function of the distance between their locations$r_{i}$ and$r_{j}$. Specifically, the element in the $i$th the row and $j$th column of the covariance matrix was chosen to obey the Gaussian-like function:

$$\Lambda\left( i,j \right)=\exp[({r_{i}-r_{j})}^{2}/d^{2}]$$

(S. 2)

where $d$ is the constant describing how fast the correlation decreases. A high value of d will lead to the assumption of roughly uncorrelated sources, whereas decreasing values correspond to the assumption of correlated sources within enlarging patches of cortical neurons. Note that, in the diagonal, the entries will always be 1, but this exact number is irrelevant since the scaling is indirectly controlled by the regularization factor $\delta$ (i.e., the assumed signal-to-noise ratio).

## 2 Supplementary results

### 2.1 Within- and between-participant test-retest reliability

On average, inter-session CCCs for the EEG responses elicited by both sham and active TMS were evidently high both within- and between- participants (Fig. S4 and Table. S1). Similar to the results at the group level, the test-retest reliability for the ‘cleaned’ TEPs decreased after removing PEPs. Notably, the within-participant CCCs remained higher than the pre-stimulation baseline EEG for all cortical targets. But the between-participant reliability of the ‘cleaned’ TEPs in mPFC was generally poor compared to AG and SMA, as indicated by the CCC values distributed around zero.

## Supplementary Figures and Figure captions


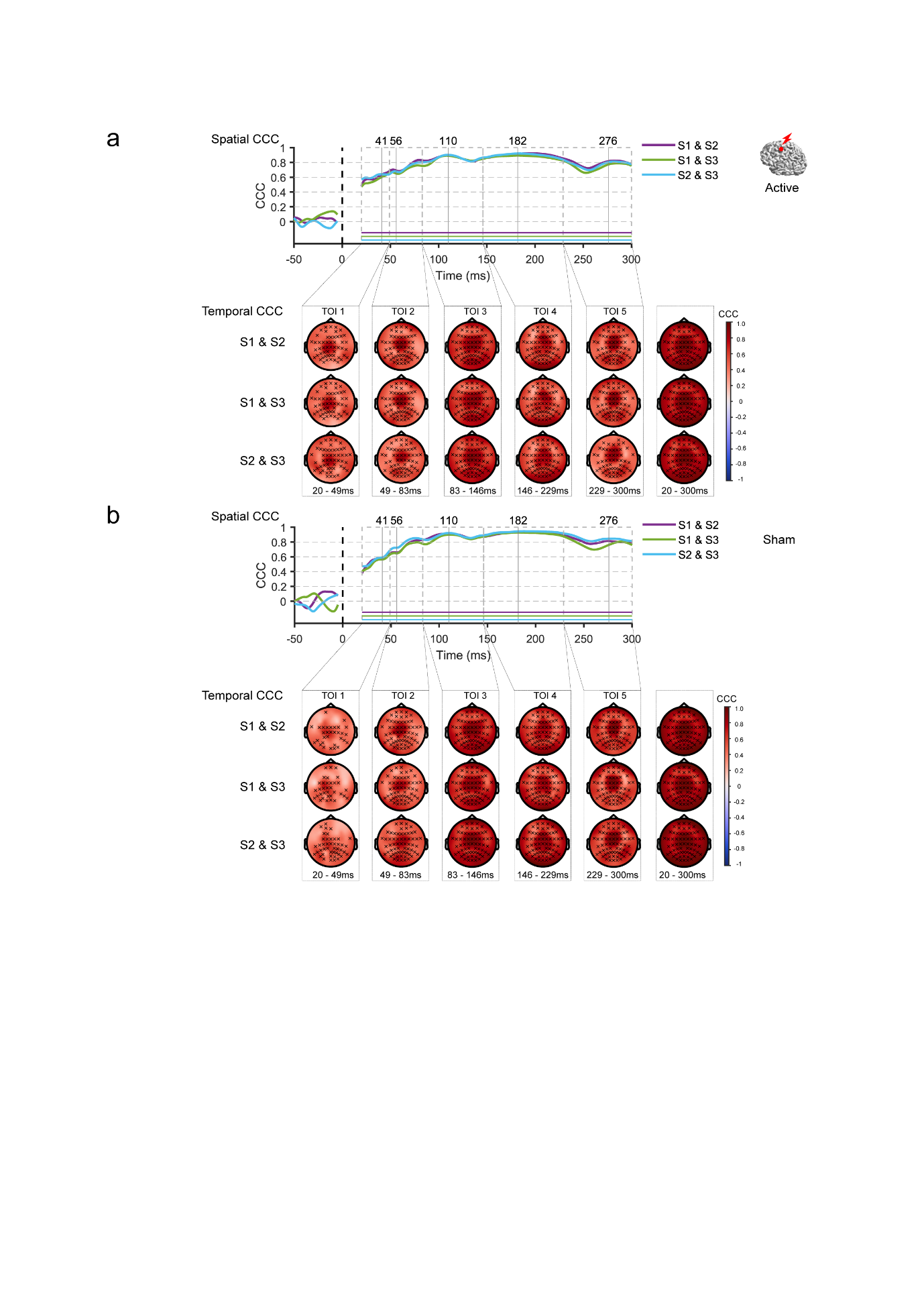


**Fig. S1** Test-retest reliability of the evoked EEG potentials by active TMS (a) and sham TMS (b) of SMA. The red dot on a template brain indicates the cortical target. The upper panel shows the spatial inter-session CCCs, with traces (purple, green, and blue) representing the group averages of CCCs for each pair of sessions. Horizontal lines indicate time points where CCCs significantly differ from zero. The lower panel displays topography of the temporal CCCs within each TOI, x indicating electrodes with CCCs significantly different from zero. Abbreviations: CCC, concordance correlation coefficient; S1, S2, S3, sessions 1-3; SMA, supplementary motor area; TMS, transcranial magnetic stimulation; TOI, time window of interest.


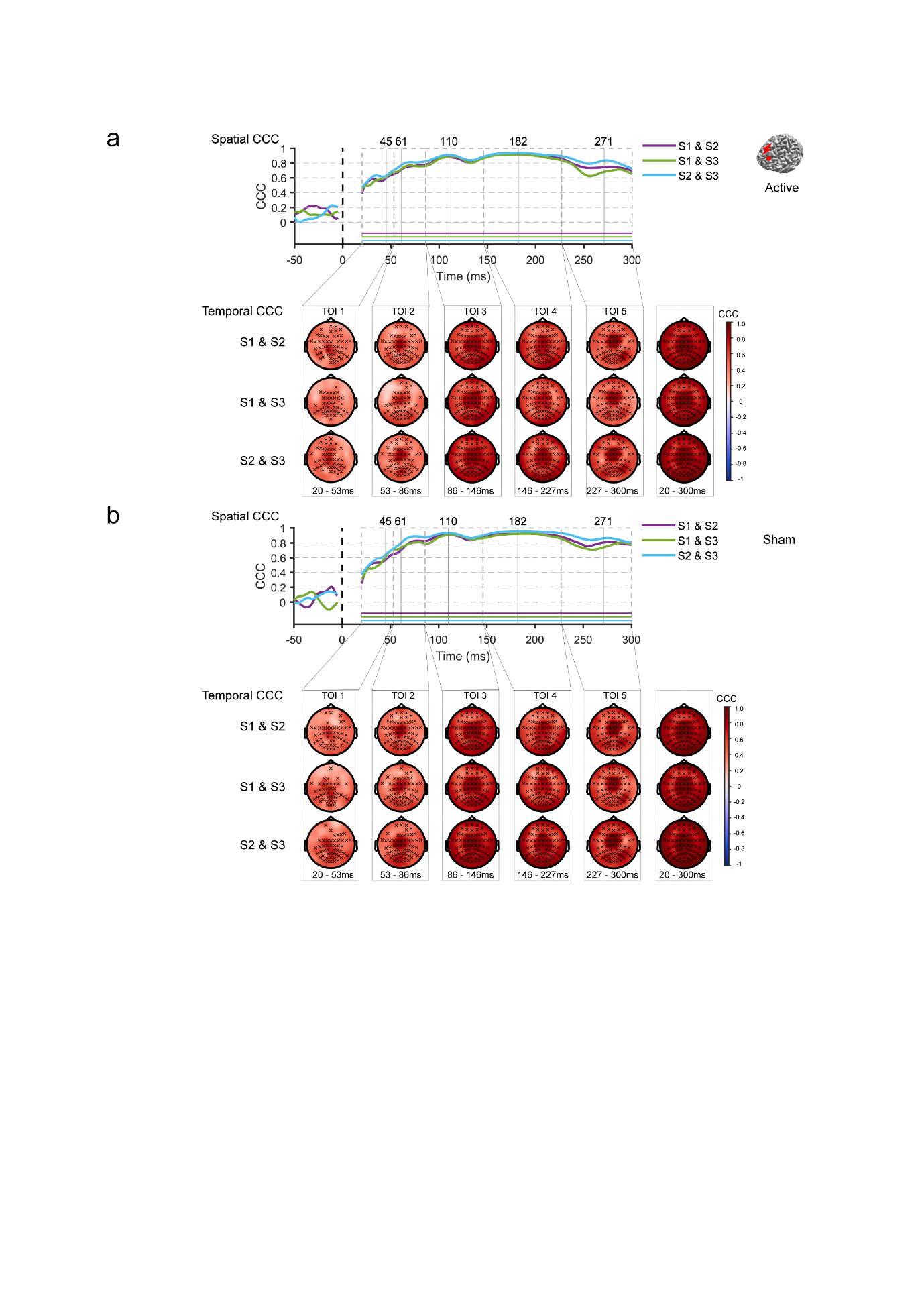


**Fig. S2** Test-retest reliability of the evoked EEG potentials by active TMS (a) and sham TMS (b) of mPFC. The red dot on a template brain indicates the cortical target. The upper panel shows the spatial inter-session CCCs, with traces (purple, green, and blue) representing the group means of CCCs for each pair of sessions. Horizontal lines indicate time points where CCCs significantly differ from zero. The lower panel displays topography of the temporal CCCs within each TOI, x indicating electrodes with CCCs significantly different from zero. Abbreviations: CCC, concordance correlation coefficient; mPFC, medial prefrontal cortex; S1, S2, S3, sessions 1-3; TMS, transcranial magnetic stimulation; TOI, time window of interest.


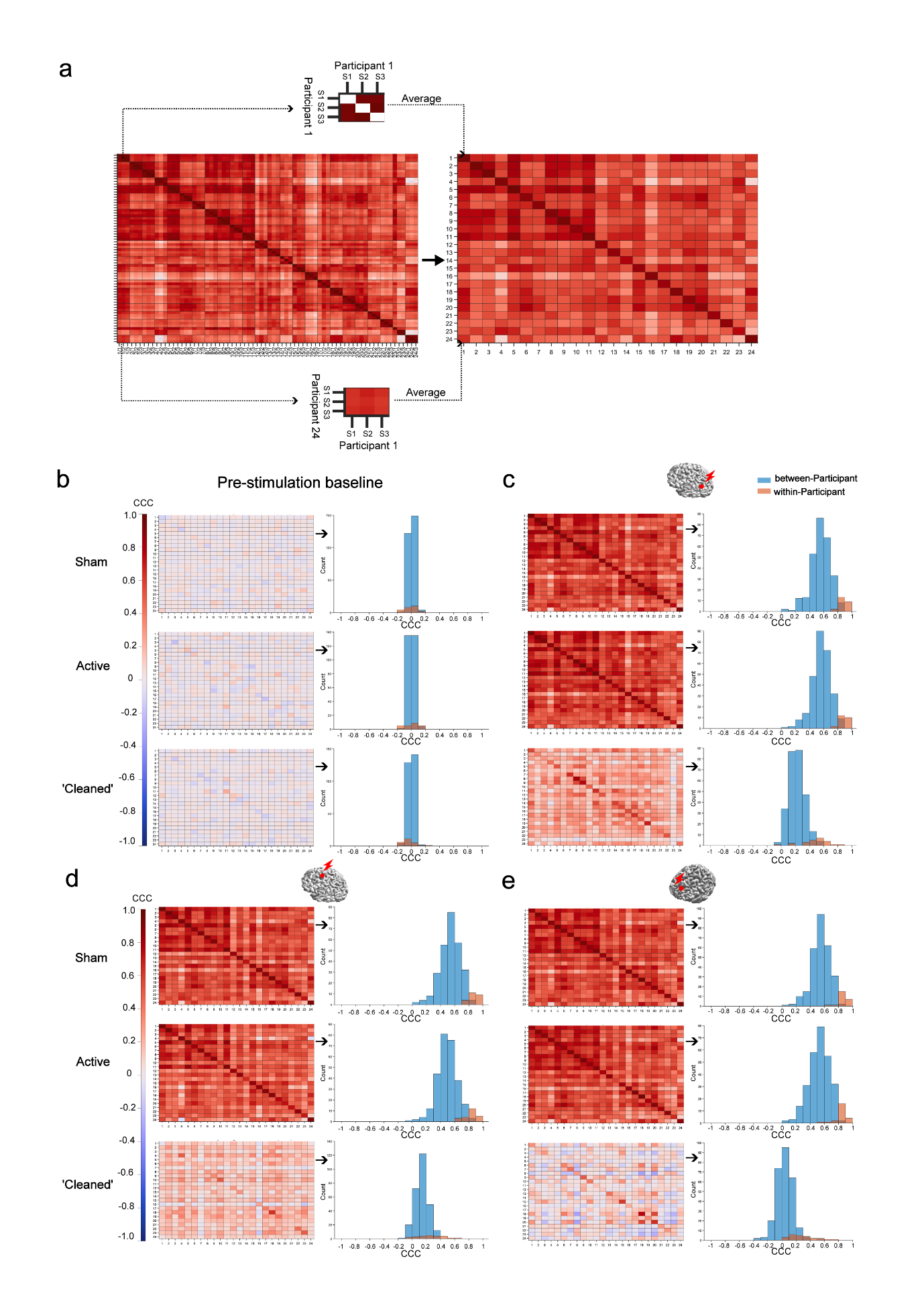


**Fig. S3** Individual-level test-retest reliability. (a) The inter-session CCC Matrices. Each entry in the matrix (72 x 72; left) represents the inter-session CCC values calculated between a specific combination of sessions and participants. The entries were then grouped by participants, and the CCC values within each block were averaged, transforming into a 24 x 24 matrix (right). The diagonal entries represent the within-participant CCCs, and the off-diagonal entries are the between-participant CCCs. Two representative blocks illustrate the transformation: the within-participant 1 CCC (excluding the diagonal), and the between participant 1 and participant 24 CCC. Within- and between-participant CCCs for the pre-stimulation baseline EEG (b) and the evoked EEG potentials by TMS of AG (c), SMA (d), and mPFC (e) in three conditions: sham (top row), active (middle row), and ‘cleaned’ (active – sham; bottom row). The red dot on a template brain indicates the cortical target. The CCC matrix and the distribution of the within- and between-participant CCCs are shown for each condition. Abbreviations: AG, angular gyrus; CCC, concordance correlation coefficient; mPFC, medial prefrontal cortex; S1, S2, S3, sessions 1-3; SMA, supplementary motor area.


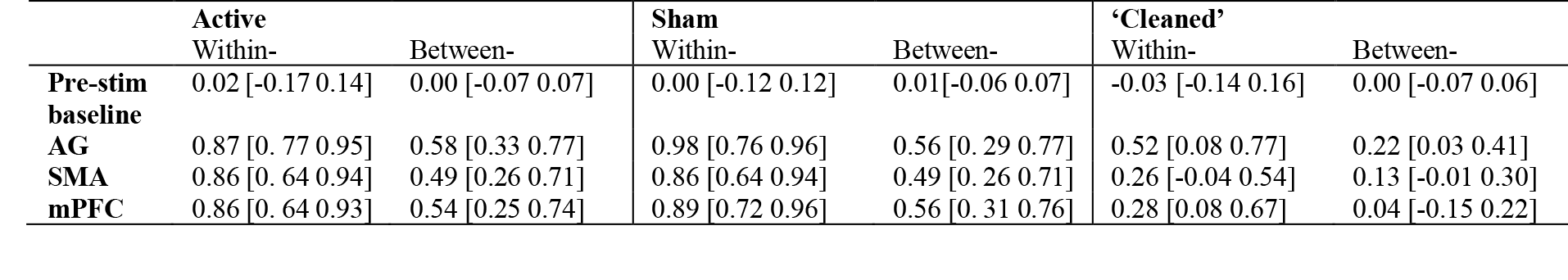


**Table S1** Within- and between- participant inter-session CCCs. NB: value in every cell every cell represents the median, and the interval from the 5th percentile to the 95th percentile (given as: “median [q(0.05), q(0.95)]”). Abbreviations: AG, angular gyrus; CCC, concordance correlation coefficient; mPFC, medial prefrontal cortex; SMA, supplementary motor area.

## Supplementary animations and animation captions

**Animation_S1**(separate file). Propagation of source activation following TMS of mPFC. The top row displays the spatial distribution from three different perspectives. The plot on the bottom right shows the time series of normalized current density, averaged within the ROI (blue) shown to the left. The solid line represents the group average, and the shaded area is the standard error. Abbreviations: mPFC, medial prefrontal gyrus; ROI, region of interest; TMS, transcranial magnetic stimulation.

**Animation_S2**(separate file). Propagation of source activation following TMS of SMA. The top row displays the spatial distribution from three different perspectives. The plot on the bottom right shows the time series of normalized current density, averaged within the ROI (yellow) shown to the left. The solid line represents the group average, and the shaded area is the standard error. Abbreviations: SMA, supplementary motor area; ROI, region of interest; TMS, transcranial magnetic stimulation.

**Animation_S3** (separate file). Propagation of source activation following TMS of AG. The top row displays the spatial distribution from three different perspectives. The plot on the bottom right shows the time series of normalized current density, averaged within the ROI (red) shown to left. The solid line represents the group average, and the shaded area is the standard error. Abbreviations: AG, angular gyrus; ROI, region of interest; TMS, transcranial magnetic stimulation.
